# Tripping on Acid: Trans-Kingdom Perspectives on Biological Acids in Immunity and Pathogenesis

**DOI:** 10.1371/journal.ppat.1003402

**Published:** 2013-07-18

**Authors:** Michael F. Criscitiello, Martin B. Dickman, James E. Samuel, Paul de Figueiredo

**Affiliations:** 1 Comparative Immunogenetics Laboratory, Texas A&M University, College Station, Texas, United States of America; 2 Department of Veterinary Pathobiology, College of Veterinary Medicine and Biomedical Sciences, Texas A&M University, College Station, Texas, United States of America; 3 Department of Plant Pathology and Microbiology, College of Agriculture and Life Sciences, Texas A&M University, College Station, Texas, United States of America; 4 Norman Borlaug Center, Texas A&M University, College Station, Texas, United States of America; 5 Department of Microbial and Molecular Pathogenesis, College of Medicine, Texas A&M Health Science Center, College Station, Texas, United States of America; International Centre for Genetic Engineering and Biotechnology, India

## Preface

Acid is fundamental to the immune mechanisms of eukaryotes. Therefore pathogens have evolved a myriad of strategies to evade, suppress, or exploit biological acids to gain access to host resources. Here, we describe several intracellular and extracellular pathogens to illustrate our current understanding of how acid plays central roles in animal and plant immunity, and also how it can be produced and exploited by microbes for pathogenic success.

## The Importance of Acid

**Figure 1:**
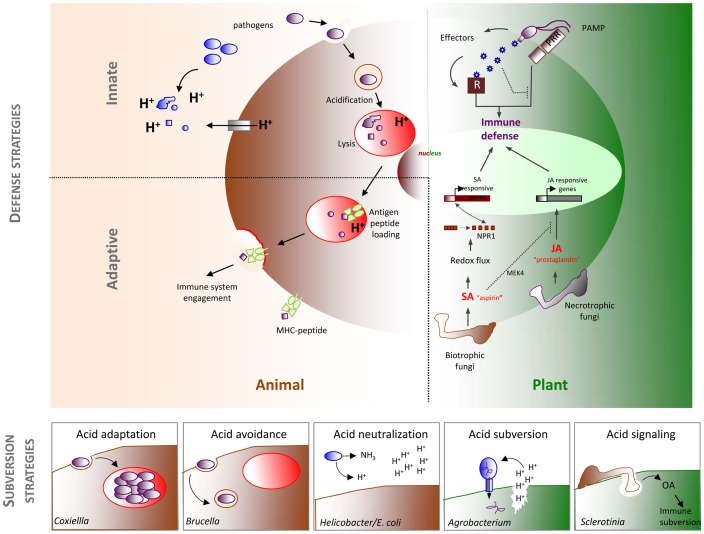
Acid is found in pathogenesis and defense in diverse symbiotic relationships. Cellular schematic shows the use of acid in innate and adaptive immunity of plant and animal cells (top). Subversion strategies of five model pathogens discussed in detail are shown in lower insets. Acid is denoted by red or H^+^. PAMP, pathogen-associated molecular patterns; PRR, pattern recognition receptors; R, (plant) resistance genes; MHC, major histocompatibility complex; SA, salicylic acid; JA, jasmonic acid; OA, oxalic acid.

The pH of an aqueous solution constitutes one of its most fundamental properties. Cells and organisms are largely aqueous entities and have evolved sophisticated strategies for sensing, exploiting, and modifying the pH of their surrounding environments for assorted biological processes, including nutrient acquisition, intercellular communication, virulence, and defense against invading pathogens. At the same time, abnormal intracellular or extracellular pH values are found in several human and plant diseases, including cancer [1] and tumorigenesis in plants. The pH of the cytosol of most eukaryotic cells is slightly alkaline, at 7.2–7.4 [2]. However, cells generate and exploit biological acids for essential functions, including the denaturation of proteins destined for degradation and the activation of acid hydrolases that mediate this process [3], and as potent activators of intracellular signaling events [4]–[7]. Here, we focus on how biological acids are generated, exploited, and manipulated by hosts and pathogens during infection and disease progression. Our analysis features a comparative approach. We consider the function and measurement of acid in several host-pathogen systems. We provide examples of how acid is used in innate and adaptive immunity, inside and outside of cells, by pathogens of medical, agricultural, and economic importance, from bacterial and fungal Kingdoms, parasitizing both plants and animals (Figure 1). We conclude by comparing broad themes that bridge or separate the mechanisms by which evolutionarily divergent pathogens and hosts evade, subvert, or exploit biological acids.

## Intracellular Acids and Their Measurement

The endocytic and phagocytic pathways of eukaryotic cells contain acidic intracellular compartments that carry out diverse functions, including nutrient acquisition [8], [9], immunological information processing [10], protein and membrane degradation [3], apoptosis [11], cellular repair and autophagy [12], and host defense [13]. Specialized functions for these pathways have often been described. In plants, for example, endocytic physiology influences gravitropism, guard cell movement, and plant hormone transport (for review, see [14]). Low pH is a critical requirement in several of these processes.

The pH of early endosomes, late endosomes, and lysosomes/vacuoles are approximately 6, 5.5, and below 5, respectively, although their pH varies with cell type, cultivation conditions, and biological context [15]. These acidic compartments communicate with one another through the vectoral exchange of membrane and protein along evolutionarily conserved trafficking pathways [10], [16]. In the endocytic pathway, extracellular proteins that are destined for internalization and degradation are captured by cell surface receptors or fluid phase engulfment. The internalized materials are sequentially trafficked from early to late endocytic compartments for delivery to acidic terminal compartments, which are designated lysosomes or terminal vesicles/vacuoles in animal and plant systems, respectively. During this process, the acidity of endocytic organelles increases. Newly synthesized proteins can also be directly delivered to endocytic compartments from the trans-Golgi network, or transported to the plasma membrane and then subsequently endocytosed. The mannose-6-phosphate receptor pathway mediates the former trafficking event [17]. Finally, contents can be delivered to acidic organelles via the autophagy pathway. In this process, membranes engulf cytoplasmic contents and/or subcellular organelles for eventual maturation and delivery to lysosomes/vacuoles, where these materials are degraded [18]. The autophagy pathway has been shown to be critical for diverse biological processes in plants, animals, and fungi, including survival during periods of nutrient limitation, (embryonic) development, apoptosis, abiotic stress, and host defense [19].

Considerable research, using yeast, insect, worm, plant, and mammalian model systems, has been performed to elucidate the mechanisms by which acidic pH is achieved within the organelles of eukaryotic cells. The acidification of organelles in the endocytic pathway has been shown to be mediated by the activity of vacuolar-type ATPase (V-ATPase) enzymes [20]. These proteins exploit ATP to drive protons into the lumen of endocytic membranes [21], [22]. In parallel with this movement of protons is the pumping of cations out of the organelle, to dissipate the development of a restrictive electrochemical gradient across the vesicular membrane [23]. Mechanisms mediating the biogenesis of acidic compartments have also been extensively investigated. In fact, the biogenesis of lysosomes and vacuoles constitutes an important subfield of cell biology [24], and several excellent reviews on this subject have recently been published [14], [25]–[27].

The task of measuring the pH of intracellular organelles presents several challenges. First, the subcellular organelles of eukaryotic cells are small (from 0.1 µm in fungi to 50 µm in plants) and provide no direct access to the outside environment once formed [28], [29]. Therefore, biochemical analysis of the lumen of these compartments requires their fractionation and isolation. Moreover, intracellular organelles are highly dynamic entities that change composition as they mature or travel along defined membrane trafficking pathways. This fact poses challenges to the analysis of heterogenous, often nonsynchronous populations of subcellular compartments. Hence, light microscopy approaches are favored for tracking organellar pH in living cells, and a variety of tools have been developed for this purpose.

Early estimates of the pH of intracellular organelles of plants, animals, and microbes were based upon the use of acidotropic dyes [30]–[33]. These molecules traverse biological membranes and accumulate in acidic intracellular organelles, thereby providing an indirect, low cost, and qualitative estimate of organellar pH. More recently, a variety of sophisticated technologies have been used to estimate intracellular pH values, including the pH of endocytic and other intracellular organelles. These technologies include pH-responsive microelectrodes [34], NMR [35], and absorbance spectroscopy [36]. However, fluorescence microscopy provides the most compelling technology for analyzing (with high sensitivity and resolution) the spatial and temporal dynamics of pH changes inside living cells. For example, probes that emit fluorescence in a manner that is dictated by their state of protonation can be used to estimate organellar pH [37]. These reagents, which include Oregon Green and engineered green fluorescent protein variants [38], also support ratiometric measurements, which can then be converted to absolute pH levels by comparison to calibration curves [39]–[41]. These ratiometric methods are also insensitive to changes in fluorescence introduced by parameters other than pH, including focal plane or photobleaching. Therefore, approaches that exploit ratiometric indicators provide a more precise, specific, and robust measure of organellar pH than their acidotropic fluorophore counterparts, like LysoTracker [42]. Finally, fluorescent ratiometric analyses of intracellular pH can be extended to investigate interactions between intracellular pathogens and host cells by labeling live pathogens with pH-sensitive dyes (carboxyfluorescein, fluorescein, or Oregon Green) so that viability is not compromised, infecting host cells with the labeled pathogens, and then performing coincident analysis of pathogen intracellular trafficking and vacuolar pH [43].

## Acids in Immune Systems

Biological acids play central roles in both innate and adaptive immune systems that dictate host-pathogen interactions. All living things possess innate immune mechanisms, while vertebrates benefit from the classically defined, lymphocyte-mediated adaptive immunity as well.

### Acid in Innate Immunity

In animal innate immunity, acid functions extracellularly and intracellularly. Low pH plays an important role extracellularly in controlling the microbial flora at mucosal sites, particularly in tetrapod vertebrates. The gastric acid of the stomach limits the range of prokaryotes that can continue down the alimentary canal to join intestinal populations [44]. The vaginal microbiome maintains an acidic environment that limits protozoan, fungal, and bacterial infections [45]. Intracellularly, biological acids and acid hydrolase enzymes mediate the killing of non–acid-adapted organisms that are phagocytosed into progressively acidifying vesicles epitomized by the lysosome. This process still provides predatory feeding and phagotrophic nutrition for some protists [46]. Pattern recognition receptors (PRR) such as the Toll-like receptors (TLR) bind perceived threats by recognition of pathogen-associated molecular patterns (PAMPs). In addition to signaling the innate immune system, PRR recognition of PAMPs in triploblastic animals can doom PAMP-bearing microbes to phagosomal degradation in acidic vesicles [47]. Moreover, several PRR such as TLR-3, TLR-7, and TLR-9 have evolved to sense nucleic acid PAMP from acidic endosomes, rather than the cell surface, taking advantage of the acidic degradation of virus and virus-infected cells by the low-pH vesicle to sense pathogen-indicative double-stranded RNA, single-stranded RNA, or DNA with unmethylated CpG dinucleotides [48]. Thus, acid is used both to selectively control microbial populations on animal surfaces by filtering them based upon acid tolerance, and to identify and eradicate those that are engulfed by patrolling leukocytes.

Plants rely on the innate immune system for defense by sensing pathogen-derived molecules for nonself recognition [49]. Plant immune receptors are known as PRR that recognize PAMPs, such as bacterial flagellin and fungal chitin as well as plant-derived signals that arise from damage caused by pathogen challenge, known as damage-associated molecular patterns (DAMPs). PAMP and DAMP recognition is ancient and shared by plants and animals. PRR binding activates broadly but moderately effective PAMP-triggered immunity (PTI). Plant pathogens have evolved mechanisms to breach this line of defense by acquisition of effector molecules that are secreted into the plant cell and perturb host immune responses by either avoiding detection or suppressing PTI signal transduction. In the continuing arms race, plants have developed a second tier of defense in which resistance gene products (R proteins) [49] mediate recognition of specific pathogen effectors and trigger effector-triggered immunity (ETI), which generally culminates in host programmed cell death. *R* gene products contain leucine-rich repeats (LRR) and nucleotide binding sites and belong to the CATERPILLAR/NOD/NLR family of proteins that mediate cytosolic PAMP surveillance in animals as well [50], thus exhibiting trans-kingdom conservation.

Organic acids are crucial messengers of effector-triggered immunity activated by *R* gene products of plants. These include the hormone salicylic acid that mediates endogenous defense signals and transmits systemic responses that orchestrate systemic acquired resistance [51]. This response is thought to be one of an antagonistic triumvirate of acid-regulated stress responses in plants, including jasmonic acid for wound healing and insect protection and abscisic acid for environmental stresses [52]. Ethylene signaling has somewhat redundant overlap with these latter two. Acids are also used in the pathogenic counterattack, as several necrotrophic (requiring dead tissue/cells for growth and reproduction) plant pathogens produce oxalic acid to control host cell death programs, as discussed in more detail below.

### Acid in Adaptive Immunity

The adaptive immune system shared by jawed vertebrates is mediated by lymphocytes and has hallmark characteristics of specificity and memory. The adaptive system enables the preemptive engineering of our lymphocyte repertoires through immunization, one of the most powerful tools for global public health in the face of infectious disease. Like the plant ETI, the adaptive system is a second line of defense behind the innate system. While slower in initial response in comparison to the innate, the adaptive system is plastic in its mitotic expansion and contraction capability, surgical in the fine molecular specificity of its targeting, and diverse in its effector mechanisms tailored to classes and locales of pathogen.

The vertebrate adaptive immune system works in conjunction with the more ancient innate system. The adaptive system may have originally evolved to manage the vast populations of commensal (better termed mutualistic) bacteria at mucosal surfaces where low pH was already in use [53]. When the relationship between host animal and symbiont turns parasitic, immune mechanisms are used to eliminate or contain the microbe and limit pathology, akin to the “hypersensitive response” of plants.

**Figure 2:**
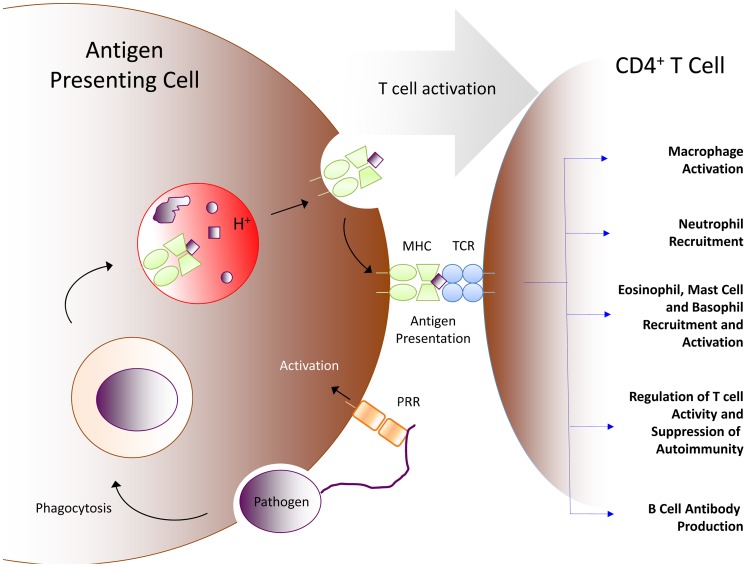
Acid's role in initiating adaptive immunity. An antigen-presenting cell activated by innate PRR will present peptide antigen generated in acidic vesicles to a helper T cell via MHC class II. Activated by this presentation of specific antigen, the helper T cell can then mediate many different immune effector functions, depending on the subtype of helper CD4^+^ T cell, context, and signals from the APC. Five such major immune effector pathways are suggested here.

Most adaptive immune responses require activation of specific helper T lymphocytes, and the low-pH phagolysosomal system is important for this activation. Unlike B cells that recognize free antigen, T cells recognize peptide antigen in the context of major histocompatibility complex (MHC) molecules. Two different classes of MHC molecules present peptide to two different classes of T cells [54]. Helper T cells are restricted to being activated only by MHC class II molecules on antigen-presenting cells (APC) that present peptide antigen. Dendritic cells are the best APC, but macrophages and B cells are also very competent in T cell activation. This APC hurdle is a major checkpoint to initiating an adaptive immune response (Figure 2). For example, macrophages harboring wily intracellular stowaways typically need “help” via cytokine and co-stimulatory signals from an activated helper T cell to in turn become activated and execute effector functions. Helper T cells can only be activated by presentation of peptide antigens specific for their somatically recombined T cell receptor gene products in the context of self-MHC class II molecules. This MHC class II–presented antigen must arise from an APC that has itself been activated by ligation of its innate PRR (such as TLR). The generation of the antigenic peptides presented by APCs is acid dependent.

**Figure 3:**
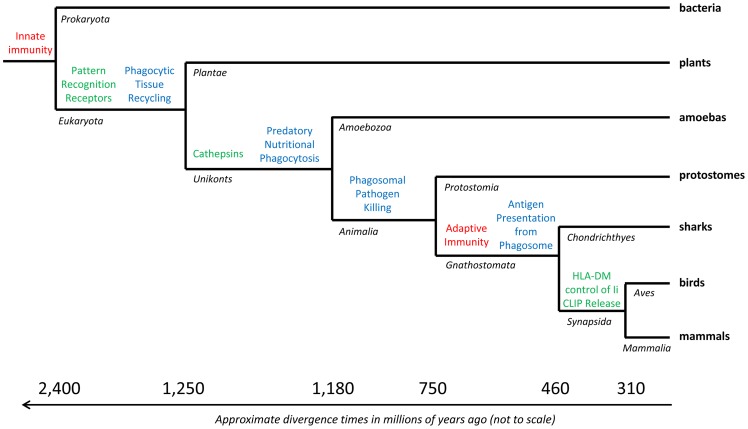
Phylogeny of acidic phagolysosome use in immunity. Simplified phylogeny of life, marking major hypothesized steps supported by current comparative biology in the co-opting of the acidic phagolysosome system in innate and adaptive immunity (blue). Sister taxon names are illustrative and not necessarily of same phylogenetic rank, and genetic distances are not to scale. All life has innate immunity, but only vertebrates have adaptive immunity (red). Origins of key proteins that regulate the system are shown in green.

The low pH of compartments of the phagolysosomal pathway is co-opted by the adaptive as well as the innate immune system for defensive purposes. We suggest that the cellular physiology of this pathway has evolved from functioning in recycling of cellular debris and tissue remodeling in early eukaryotes [46], to functioning primarily for nutritional purposes in heterotrophic protozoa, on to the killing of pathogens in innate immunity [55], and finally to elegant regulation of adaptive immunity via antigen processing [56] (Figure 3). Jawed vertebrates use a sophisticated antigen processing pathway to load MHC class II with peptide antigen from the acidified phagolysosome. Newly synthesized class II α and β chains assemble in the pH-neutral endoplasmic reticulum [57] together with a glycoprotein called the invariant chain [58], [59]. An important role of the invariant chain is to keep MHC class II from being loaded prematurely with peptide from the endoplasmic reticulum or Golgi, retaining that place for products of the acidified phagolysosomal system.

**Figure 4:**
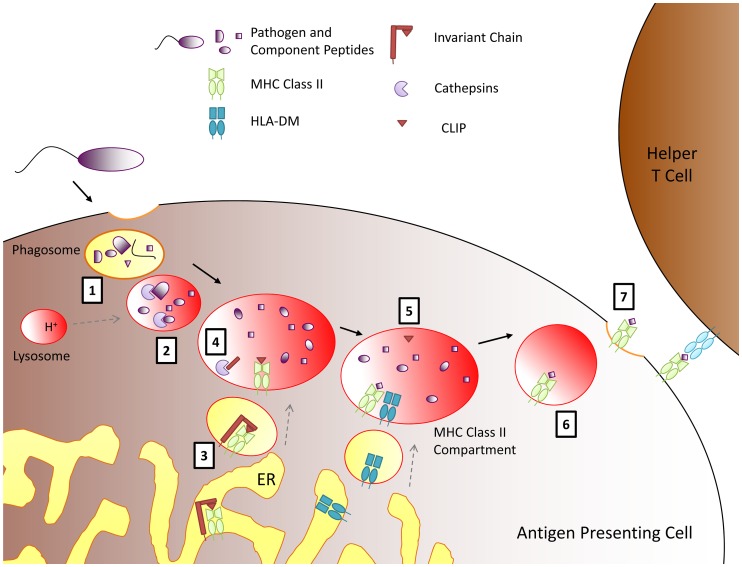
Acid-active cathepsins cleave phagolysosomal antigens in the MHC class II pathway. Phagocytosed antigens are degraded to peptides (grey) by acids and acid-active cathepsin proteases as the endosomal pH decreases due to fusion with lysosomes (1). During their trafficking from the ER to the cell surface, MHC class II molecules (light green) pass through these acidified vesicles (2). Invariant chain (red) chaperones MHC class II from the ER to an acidified endosome, all the while protecting the peptide binding groove of MHC class II from premature loading (3). Invariant chain is cleaved by cathepsins but leaves the CLIP portion (red triangle) in the MHC peptide binding site (4). In a specialized late endosome, the MHC homolog HLA-DM finally binds to the MHC class II/CLIP complex and releases CLIP (5), allowing other peptides to bind before the MHC class II travels to the cell surface (6). There it can present antigen to T cells (7).

In the trans-Golgi, the MHC class II/invariant chain complex is diverted from the secretory pathway to the endocytic pathway (Figure 4). In the low pH of the MHC class II compartment, cathepsin proteases are activated to further cleave the invariant chain, leaving only CLIP (the class II–associated invariant chain peptide) in the peptide binding cleft [60]. HLA-DM can then bind class II and catalyze the release of CLIP, facilitating its exchange for phagolysosomal antigenic peptides before MHC transport to the cell surface. Pathogen blockade (e.g., by the human immunodeficiency virus) of the progressive cleavage of the invariant chain results in the accumulation of invariant chain intermediates, constipation of the antigen presentation pathway, and immunoevasive reduced surface expression of MHC class II [61].

Cathepsins, related to papain, are the crucial acid-activated proteases that cleave the invariant chain and also degrade lysosomal contents to peptides appropriate for antigen to be loaded in MHC class II [62]. The cathepsins primarily involved in antigen processing are L and S. Although invariant chain and cathepsins of adaptive immunity likely evolved with the jawed cartilaginous fishes (gnathostomes, see Figure 3) [63], comparative phagosome proteomics show acid-active cathepsins to be an original and fundamental component of the ancestral eukaryotic phagolysosomal system [64].

Thus, the acidic phagolysosomal system prepares peptide antigens for the initiation of most cellular adaptive immune responses, mediated by the MHC class II system of presentation to helper T cells.

## Acids in Host-Pathogen Interactions

Given the role that acid plays in host defense and antigen presentation in animal pathosystems, it is perhaps not surprising that both plant and animal pathogens have evolved sophisticated systems for adapting to, avoiding, or subverting the threats that acidic environments and acid-mediated defense processes pose. We illustrate this point using two intracellular bacterial pathogens—*Coxiella burnetii* and *Brucella melitensis*—which have evolved disparate strategies for adapting to life in acidic environments and avoiding killing by acidic organelles. Two extracellular mucosal pathogens—*Helicobacter pylori* and *Escherichia coli*—exemplify tactics used to colonize the extreme pH of the alimentary canal. In addition, we describe the way in which the plant pathogens *Agrobacterium tumefaciens* and *Sclerotinia sclerotium* exploit and produce acidic environments, respectively, to promote their pathogenic programs. These host-pathogen systems have been chosen both for their position as major models in which acid defense mechanisms have been elucidated as well as their importance in human, animal, and plant pathology.

### Intracellular Acid

Many organisms generate and use acidic environments to thwart infection by pathogens. However, some pathogens have evolved elegant strategies to defeat these acidic defense mechanisms, including the ability to withstand or thrive in highly acidic environments. The intracellular pathogen *Coxiella burnetii* provides a compelling example of such an adaptation. The Gram-negative bacterium *C. burnetii* is the causative agent of Q fever. Its natural reservoir in the United States consists mainly of dairy cattle, sheep, and goats [65], and its extreme infectivity (single bacterium [66]) led to its weaponization before the United States' biological warfare program was terminated in 1969 [67].

**Figure 5:**
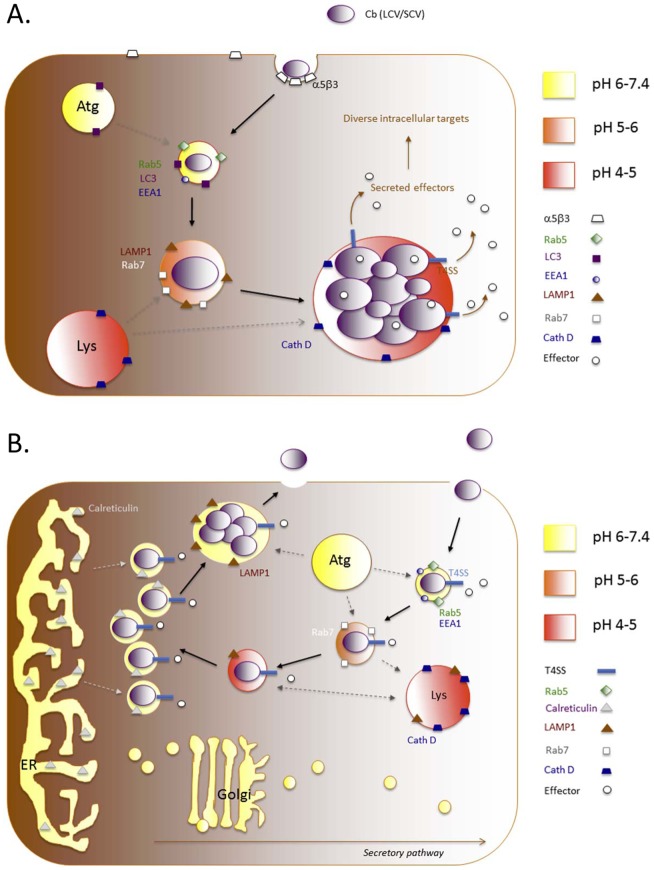
*Coxiella* and *Brucella* use distinct mechanisms for intracellular pathogenesis. **A.** *C. burnetti* thrives in the acidic phagolysosome system, requiring low pH for the transition from quiescent small cell variants (SCV) to metabolically active large cell variants (LCV). Several of the transmembrane proteins that mark the *Coxiella*-containing vacuole (CCV) through this transition are shown. The Dot/Icm type IV secretion system is used by *C. burnetti* to deliver proteins into the host cytosol [138], and renovate the lysosome into a CCV [139]. Cb, *Coxiella burnetti*; Atg, autophagosome; Lys, lysosome. **B.** Working model of *Brucella* intracellular parasitism. *Brucella-*containing vacuoles avoid fusion with acidic lysosomes, and instead traffic to a compartment that is decorated with ER markers for replication. The Type IV secretion system (T4SS) of the pathogen is critical for appropriate trafficking, and mutants that harbor mutations in the T4SS traffic to the lysosome where they are killed. Several T4SS secretion substrates have been identified, and it has been postulated that these molecules contribute to supporting the intracellular lifestyle of the pathogen. Replicative *Brucella* can exit cells by trafficking along a pathway that involves selective interactions with components of the host cell autophagy biogenesis machinery. Approximate vesicular/vacuolar pH is indicated by color, and the Golgi is generally more acidic than the ER [57, 140–142].

*C. burnetii* replicates within phagolysosome-like *Coxiella*-containing vacuoles (CCVs) and is dependent on the low pH of this compartment to activate a developmental process that turns metabolically quiescent small cell variants (SCV) into their metabolically active, replicating large cell counterparts [68] (Figure 5A). *C. burnetii* is taken up via complement receptor 3 and α_v_β_3_ integrin-mediated mechanisms into macrophages [69]. Actin-dependent phagocytosis leads to trafficking through an early endosome, progressing to late endosome and the phagolysosome-like terminal CCV [70], [71]. The trafficking appears slightly delayed compared with latex bead uptake [72], and membrane markers for Rab5, Rab7, Rab24, microtubule-associated protein-1 light chain alpha 3 (LC3), lysosomal-associated membrane proteins (LAMP)-1, LAMP-2, and LAMP-3, and flotillin 1 and 2 progressively decorate the CCV [73]–[75]. Connection to the autophagosome compartment appears essential to support the biogenesis of a compartment that can support productive replication of the pathogen [74], [76]. In addition, CCV membrane development to a spacious vacuole requires access to continual host-derived cholesterol biosynthesis [77], [78] and connection to a canonical secretory pathway [79].

Several lines of investigation support the hypothesis that maintenance of the acidic pH of the spacious vacuole is essential for *C. burnetii* replication. First, studies using ratiometric, pH-sensitive probes have demonstrated that *C. burnetii* replicates in vacuoles that possess an acidic pH [80]. Inhibition of host vacuolar ATPase activities that maintain the acidic pH of late endocytic and lysosomal compartments by treatment with bafilomycin significantly impairs the intracellular replication of the pathogen. Similarly, neutralization of vacuolar pH by treatment with the chaotropic agent chloroquine inhibits intracellular replication [81]. Taken together, these data indicate that the *C. burnetii* replicative niche has an acidic pH, and raise many questions about the mechanisms by which *Coxiella* and other acid-adapted intracellular pathogens survive in the highly acidic environment of the CCV terminal compartment [82], [83].

Several strategies have been proposed to contribute to acid tolerance of *C. burnetii*, and the development of axenic growth conditions and suicide plasmid-based targeted gene deletion methods is allowing identification of virulence mechanisms and genes in this model [84]. First, the intracellular trafficking of the pathogen along the endolysosomal pathway pauses immediately after entry [72]. This pause in phagolysosomal maturation has been hypothesized to play a critical role in acid adaptation by providing the pathogen with sufficient time to prepare (through the expression of acid tolerance factors) for the onslaught of acid that follows. Second, stress-response and vacuole-detoxification genes are dramatically upregulated when the pathogen invades host cells, thereby supporting its adaptation to the harsh vacuolar environment [85]. Finally, *C. burnetii* encodes an unusually high number of basic proteins. The average pI value for all predicted proteins in the genome of the Nine Mile reference strain is 8.25 [86], [87]; 60% of the proteome is acidic [88]. Moreover, approximately 45% of *C. burnetii* proteins were found to have a pI value of ≥9, which is higher than the sequenced genomes of other intracellular bacterial pathogens [89]. Orthologous products of the *RpoS* genes of *E. coli* (pI 4.6) and *C. burnetti* (pH 9.6) serve as striking examples of the extreme acid adaptation of a protein while maintaining a conserved function. It is hypothesized that *Coxiella's* skew toward production of basic proteins provides a proton sink to buffer those protons that enter the cytoplasm [88], [90]. While the low pH of the phagolysosome is a crucial parameter, *Coxiella* tolerates cathepsin and other acid protease action while thriving in this environment. *Coxiella* therefore illustrates how pathogens can adapt to acidic compartments.

In an alternative strategy, intracellular bacterial pathogens can address the threat that acidic intracellular compartments pose by minimizing their interactions with them. The intracellular bacterial pathogen *Brucella* spp. provide excellent models to analyze this strategy for addressing the threat that intracellular acidic vacuoles pose to invading pathogens. *Brucella* spp. are causative agents of brucellosis, a zoonosis of global importance [91]. In humans, the disease causes severe, debilitating, and protracted symptoms, and affects practically every organ system of the body. Brucellosis often presents in the clinic as an undulant fever [92]. However, chronic infections are frequently associated with osteoarticular disease with neurological complications. The reproductive system is also a common site of infection, and infection during pregnancy may increase the risk of spontaneous abortion. Consumption of unpasteurized milk products from infected animals is the most common route to human infection [92]. However, *Brucella* is highly infectious and can be readily transmitted in aerosolized form [93]. Brucellosis has eluded systematic attempts at eradication, even in most developed countries, and no human vaccine is available [94]. These features contribute to the classification of *Brucella* as a potential bioterror agent, and to the interest the biosecurity and world health communities have expressed in this organism.

During their intracellular trafficking within host cells, *Brucella*-containing vacuoles (BCVs) interact but avoid fusion with the host lysosome [95], [96] (Figure 5B). Instead, replicative BCVs become decorated with markers for ER [97]. Mutant strains harboring defects in the Type IV secretion system do not avoid fusion with acidic lysosomal compartments, and instead are rapidly killed after fusion with this organelle [98]. In mouse models of brucellosis, the organism persists for months in the lymph nodes and spleen [99]. However, colonization by even the most virulent strains becomes undetectable with time [100]. The persistence of this organism in the ER of host APCs may be crucial for chronic infection. Thus, avoiding the harsh environment of the host cell's acidic degradative organelles is critical to the survival and replication of this intracellular bacterial pathogen.

### Extracellular Acid in Animals

Several notable microbial pathogens, including those that infect the gastrointestinal or urogenital tracts of humans and animals, exploit or create extracellular acidic environments to promote their pathogenic programs. For example, pathogenic *E. coli* (strain 0157:H7), *Vibrio cholerae*, *Vibrio vulnifus*, *Shigella flexneri*, and *Salmonella typhimurium* are found in neutral pH environments (on food products or in water). However, after ingestion by their mammalian hosts, these pathogens encounter the severe acidic environment of the stomach (pH = 2 or lower) or urogenital systems, which normally constitute important barriers to infection by non–acid-adapted organisms. These parasites, however, have evolved sophisticated and divergent strategies to tolerate these harsh acidic environments.

A significant human pathogen that provides an understanding of an alternative strategy by which an extracellular acidic niche can be occupied is *Helicobacter pylori*, the agent whose colonization is associated with chronic gastrointestinal diseases ranging from dyspepsia to gastric and duodenal ulcers to gastric carcinoma [101], [102]. This organism chronically infects the stomach, surviving the very low pH of the lumen, burrowing into the mucus with flagella to attach to and occasionally invade epithelial cells. Survival in this environment is dependent on expression of copious amounts of urease, converting urea into buffering ammonia plus carbon dioxide [102]. In this strategy, *H. pylori* maintains a periplasmic pH at ∼6.1, while the extracellular environment can be as low as 2.0. The reaction products of urease apoenzyme (*ureA* and *ureB*) are driven specifically to the periplasmic compartment by a protein (UreI) encoded in a gene cluster with urease. UreI is a pH-gated inner membrane urea channel allowing for efficient transit to the cytoplasm of substrate (urea) and periplasmic release of reactant NH_3_, which is rapidly converted to NH_4_ by membrane-bound α-carbonic anhydrase. The transcriptional expression control, post-transcriptional recruitment, and enzymatic activity of *H. pylori* urease is optimized to function in a microenvironment of pH 3.5–∼6.0 [103].

*H. pylori* is specialized for the extreme pH of the stomach, but enteric species survive passage through the stomach for colonization of the lower gut. Many *E. coli* efficiently colonize the intestine, and provide a complementary window for understanding how pathogens address the challenge of acidic environments. The acid stress response systems of Gram-negative enteric pathogens are both enzyme and chaperone based, and include resistance pathways that exploit glutamate-, arginine-, and lysine-decarboxylase enzymes [104]. *E. coli* contains five acid resistance pathways (AR1–5), which work in concert to resist the highly acidic environment that the pathogen encounters in the gut [105]. In the AR2 system of *E. coli*, for example, the pyridoxal 5′ phosphate (PLP)-dependent GadA and GadB decarboxylases convert glutamate to gamma-amino butyric acid (GABA) and carbon dioxide (CO_2_) in a reaction that consumes a cytoplasmic proton [106]. The inner membrane antiporter GadC then transports GABA out of the cell in exchange for additional glutamate [106]. Thus, the pathogen mitigates acid stress by promoting the net export of protons outside of the cell at the expense of intracellular glutamate. Analogous systems that exploit arginine and lysine also contribute to maintaining the pH homeostasis of the cytoplasm.

Thus gut pathogens use a variety of active biochemical systems to maintain periplasmic and cytoplasmic pH at tolerable levels amidst the low luminal pH of the gastrointestinal tract.

### Extracellular Acid in Plants

Analysis of interactions between plant pathogens and their host plants provides additional insights into the role of acid in host-pathogen interactions that cannot be fully appreciated by the exclusive analysis of animal systems. The bacterial pathogens *Agrobacterium tumefaciens* and *Erwinia amylovora*, the causative agents of crown gall disease in diverse dicotyledenous plants and fire blight in apples, pears, and Rosaceous crops, respectively, induce disparate and nonoverlapping disease symptoms in plants. Nevertheless, these pathogens respond to environmental acids by modulating their virulence programs, and thus provide ideal illustrations of an important mechanism by which pathogens of both plants and animals respond to acidic extracellular environments in the context of the host-pathogen interaction.

In the *Agrobacterium* system, environmental acids drive the induction of the pathogen's virulence program. Specifically, the acidic environment created by wounded plant tissues, as well as plant-derived phenolic compounds present there, activate the expression of bacterial virulence genes (*vir* regulon) on the tumor-inducing (Ti) plasmid [107]. This activity, in turn, drives the assembly and transfer of the *Agrobacterium*-derived T-DNA from the bacterial pathogen to host cells. The T-DNA integrates into the host plant genome, triggering factors that mediate the generation of a tumor (gall) in plants. Interestingly, the acidic conditions that initiate the T-DNA virulence program of *Agrobacterium* elicit two additional and distinct responses in the pathogen—a conserved response associated with the adaptation of the pathogen to environmental acidification, and the intricate response that regulates the establishment of a stable, long-term plant-pathogen interaction [108]. Genes induced by the former response, such as the motility gene *flaA* and the heat-shock protein *ibpA*, are highly conserved and play corresponding roles in acid adaptation in other microbial systems, including in the bacterial pathogens of animals (e.g., *E. coli*, *Salmonella* spp.) [108]. An important component of the latter response is regulated by the activities of acidic plant hormone signaling molecules, including salicylic acid (SA), indole-3-acetic acid (IAA), and gamma-amino butyric acid (GABA) [109] (more on plant hormones in the following section). These plant acids generally signal through biochemically distinct and independent bacterial pathways to function additively to shut off the *Agrobacterium* virulence program and activate the quorum-quenching machinery, which promotes the establishment of a stable host-pathogen interaction. However, signal input from one pathway (an environmental stress response signaled through abscisic acid during a drought, for example) can inhibit the activation of another (such as a jasmonic acid signal for wound repair stimulated by herbivory). The activation of quorum-sensing machinery as part of the process of establishing a stable host-pathogen interaction represents a conserved theme in the virulence programs of many bacterial pathogens [110].

Extracellular acidic environments can also influence the virulence of fungal pathogens of plants, including *Ustilago maydis* [111], *Fusarium oxysporum* [112], and *Sclerotinia sclerotiorum* [113], the causative agents of tumorigenic corn smut, *Fusarium* wilt, and white mold diseases of all broadleaf plants, respectively.

### Plant Hormones

Analogous to animal hormones, plant hormones play key roles in the control of development, growth, reproduction, and, of relevance for this discussion, the regulation of immune responses to microbial pathogens. Of the principal plant hormones, five are acids, including: salicylic acid (SA), jasmonic acid (JA), abscisic acid (ABA), indole acetic acid (IAA), and gibberellic acid (GA) [114]. SA and JA and their derivatives are structurally and functionally akin to aspirin and prostaglandins, respectively. SA and JA are recognized as major defense hormones with the classical view that SA is effective against biotrophic pathogens and JA against necrotrophs, although there are exceptions [52]. Moreover, crosstalk occurs between these hormones that is often antagonistic; elevated biotroph resistance (SA) results in elevated necrotroph susceptibility and vice versa.

Systemic acquired resistance (SAR) is induced in the plant by SA following pathogen challenge. When established, SAR affords long-lasting and broad-spectrum resistance including uninfected tissue. SA levels increase following initial pathogen attack and are strongly correlated with establishment of SAR [115]. This observation is strengthened by the fact that plant treatment with exogenous SA or biologically active chemical analogs leads to SAR. Moreover, blocking SA synthesis inhibits SAR [116]. Considerable effort has been undertaken to identify the regulatory pathways mediating SAR. Of note, mutants of the *npr1* locus were found to prevent SA signaling [117]. In the uninduced state, cytosolic NPR1 is present as an oligomer via intermolecular disulfide bridges. Following SA-mediated SAR induction, alterations in cellular redox result in a reduced state leading to disassociation of the complex and release of monomeric species. Monomeric NPR1 translocates to the nucleus, interacts with the leucine zipper transcription factor TGA1 that binds to promoters of SA-responsive genes, and activates defense gene expression [118].

The jasmonate family comprises lipid-derived metabolites synthesized via the oxylipin pathway [119]. Upon synthesis, JA can be metabolized to methyl jasmonate or conjugated to amino acids [120]. Most JA responses are mediated by the F-box protein coronatine insentive 1 (COI1). *Coi1* mutants are more resistant to bacterial pathogens and show elevated SA levels [121]. In accordance with SA-JA antagonism, *coi1* plants are more susceptible to several, but not all, necrotrophic fungi. Exogenous application of JA induces broad changes in transcription patterns—in particular, of genes regulated by MYC2, a basic helix loop helix transcription factor [122]. Genetic studies revealed a family of 12 jasmonate ZIM-domain–containing (JAZ) proteins that repress JA signaling. JAZ proteins can homo- and heterodimerize *in vitro*, suggesting a possible mechanism for fine-tuning signaling responses [123]. Binding of conjugated JA or coronatine to SCF^COI1^ promotes ubiquitination of JAZs leading to proteasome degradation, relieving repression of MYC2, and facilitating activation of JA-responsive genes [124]. Alternative splicing of the c-terminal JAS domain of JAZ proteins results in reduced ubiquitination and thus reduced degradation.

Although JA is central to modulating defense against necrotrophic pathogens, it is increasingly important in other aspects of plant-pathogen interactions including SAR [125]. Once the JA pathway is activated (e.g., after wounding), a similar JA response can be triggered in distal undamaged parts of the plant. The antagonism between SA and JA signaling pathways in plants show a dramatic resemblance to the effect of the anti-inflammatory drug aspirin, which is an acetylated form of SA, on prostaglandins. Prostaglandins are structurally related to JA, and these hormones function at sites of infection or injury. SA/JA crosstalk shows similarities with the inhibitory effect of aspirin to prostaglandins in mammalian cells; however, the molecular bases of these interactions are not identical [126].

The phytopathogenic fungus *Sclerotinia sclerotiorum* provides an informative model to illustrate acid-dependent pathogenic development. This fungus produces copious amounts of the dicarboxylic acid oxalic acid (OA) as part of its virulence arsenal, but also uses OA to reprogram host signaling and modulate programmed cell death to coordinate effective pathogenesis [113].

*Sclerotinia* is an economically important necrotrophic fungal pathogen of plants with an extremely broad host range (all broadleaf dicot plants). The primary component for pathogenic success is the production and secretion of oxalic acid by the fungus [127]. Oxalic acid is found in plants, animals, and humans and is the endpoint of several metabolic processes such as the breakdown of glyoxylic acid or ascorbic acid [128], [129]. In mammals, oxalic acid plays an important role in kidney stone formation as a counter ion of calcium-forming oxalate crystals [130].

**Figure 6:**
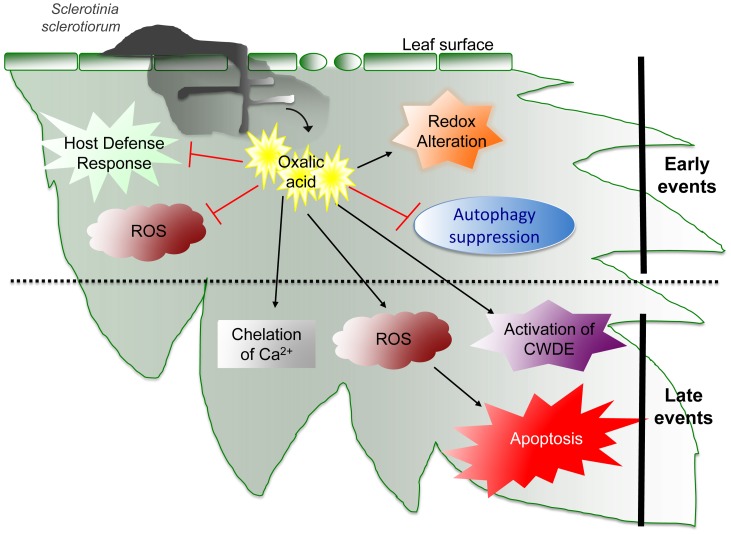
Stages of *Sclerotinia* pathogenesis. Early steps of infection create a reducing environment that dampens host defense responses and inhibits reactive oxygen species (ROS). This allows the fungal pathogen to establish and damage host tissues with cell wall degradative enzymes (CWDE). When eventual apoptotic cascades are induced, recognition occurs but too late for the host plant to prevail. (adapted from [143]).

In fungi, this “simple” organic acid is remarkably multifunctional and contributes to numerous physiological and pathogenic processes [131] (Figure 6). Oxalate-deficient mutants of *S. sclerotiorum* are nonpathogenic on all host plants tested and are also unable to develop sclerotia—highly melanized, durable overwintering structures. Oxalate secretion might enhance *Sclerotinia* virulence in several ways. Many fungal enzymes secreted during invasion of plant tissues (e.g., pectinases) have maximal activities at acidic pH and have been shown to be activated by OA. A *Sclerotinia* MAP kinase has also been identified and characterized [132], and has been shown to be required for sclerotia formation in *S. sclerotiorum*. Gene expression of this Erk-like MAPK (SMK1) is triggered by acidic pH mediated by OA; if acidification does not occur, pathogenic development is blocked [133], [134].

OA has several other deleterious effects upon the plant. It can degrade or weaken the plant cell wall via acidity and/or chelation of cell wall Ca^2+^. Oxalic acid crystals are sequestered in vacuoles, and when decompartmentalized during infection, these crystals can plug the vascular system. Oxalic acid in and of itself is directly toxic, functioning as a non–host-specific phytotoxin. While these features are correlated with fungal disease development, they do not account for wild-type pathogenesis [135].

OA can function as an elicitor of apoptotic-like plant programmed cell death, involving the modulation of the host redox environment [136]. The induction of apoptosis and disease requires generation of reactive oxygen species (ROS) in the host, a process triggered by fungal-secreted OA. Curiously, the programmed cell death process mediated by OA is independent of acidification; a direct correlation between DNA laddering, ROS induction, and cell death were all observed at neutral pH. When acidification via OA occurs, cells also die but in a mechanistically different way: necrotically without hallmarks of apoptosis such as plasma membrane blebbing and chromosomal DNA fragmentation. The specificity of this interaction is further supported by the observation that DNA fragmentation is specific to OA, as other acids such as citric acid, succinic acid, and hydrochloric acid do not induce DNA ladders [137]. DNA cleavage also was independent of oxalate formulation because OA, potassium oxalate, and sodium oxalate all caused DNA laddering, thus suggesting that programmed cell death induction is not due to the acidic nature of oxalate but rather to a property of OA itself.

Conversely, during the initial stages of infection, OA also dampens the plant oxidative burst—an early host response generally associated with plant defense. Experiments using a transgenic redox-regulated GFP reporter show that, initially, *Sclerotinia* (via OA) generates a reducing environment in host cells that suppresses host defense responses including the oxidative burst. Once infection is established, however, this necrotroph induces the generation of plant ROS leading to PCD of host tissue, the result of which is of direct and sole benefit to the pathogen. In contrast, a nonpathogenic, OA-deficient mutant failed to alter host redox status and induced autophagy and restricted growth. These results indicate active recognition of the mutant by the plant and further point to the importance of cell death control in mediating host-pathogen interactions.

Taken together, these data suggest that *Sclerotinia* establishes reductive conditions that dampen the host oxidative burst and suppress defense responses [135]. This scenario buys precious time, allowing for unimpeded fungal growth and establishment. When the plant eventually senses the presence of nonself, it is too late; the fungus is already inducing the programmed cell death of host cells.

## Conclusions and Perspective

Just as pH is a fundamental property of living systems, acid is employed by both host and pathogen in their ever-escalating arms race. On the side of the host, acid is used in a variety of mechanisms, including extracellular innate immunity at mucosal surfaces and extreme pH in the case of the mammalian stomach. Yet some pathogens have evolved mechanisms to defeat the natural defense that the acidic environment provides, exemplified by *Helicobacter*'s urease system that keeps its periplasm close to neutral and *E. coli*'s glutamate decarboxylase pathway that removes cytoplasmic protons as a component of its acid response. Acid is important in plant extracellular immunity as well and is a characteristic of wounding, but *Agrobacterium* senses this drop in pH and counters with acid-based signaling programs to ramp up and attenuate virulence effectors. Fungi such as *Sclerotinia* use OA to subvert host defense and co-opt host signaling pathways, triggering cell death via apoptosis of host cells. Thus fungal (OA) induced metabolic reprogramming of the host results in apoptosis, providing nutrients exclusively for the benefit of the necrotrophic organism.

The adaptive immune system of vertebrates pirated the acidic phagolysosomal system for not only pathogen killing but also for antigen presentation to T cells. Vesicular acid and acid-activated proteases such as cathepsins are critical to antigen processing for MHC loading. But some microbes have evolved mechanisms to cope with the low pH of vacuoles, such as *Coxiella*, while others have managed to reprogram the vesicle trafficking to mitigate interactions with harmful acidic organelles (e.g., lysosomes), as does *Brucella*. Ratiometric dyes have been crucial in facilitating the analysis of these intracellular organelles and the pathogenesis that occurs there. Table 1 lists additional pathogens that exploit or evade acid beyond the scope of this review.

**Table 1:** Notable pathogens evolved for acid management or evasion.

Mechanism	Species
Phagolysosome adaptation	*Coxiella burnetii*
Phagolysosome evasion	*Brucella melitensis*
	*Mycobacterium tuberculosis*
	*Listeria monocytogenes*
	*Toxoplasma gondii*
	*Chlamydia trachomatis*
	*Legionella pneumophila*
Adaptation to extracellular acidic mucosa	*Helicobacter pylori*
	*Escherichia coli*
	*Vibrio cholerae*
	*Trichomonas vaginalis*
	*Shigella flexneri*
	*Campylobacter jejuni*
	*Clostridium perfringens*
	*Salmonella enterica*
	*Shigella dydenteriae*
	*Neisseria gonorrhoeae*
	*Treponema palladum*
	*Yersinia enterocolitica*
	*Clostridium difficile*
	*Staphylococcus aureus*
Adaptation to plant acidic environments	*Agrobacterium tumefaciens*
	*Sclerotinia sclerotium*
	*Erwinia amylovora*

From the perspective of natural history, we see the mechanisms by which organisms exploit or subvert biological acids as central to their evolutionary success. Thus acid is one of a small, select number of fundamental biological arenas in which organic life has long fought. For example, membranes create a defined space for orderly biochemistry where metabolism and other necessities can occur protected from the chaos beyond the phospholipid bilayer. Naturally, immune systems evolve enzymes such as lysozyme, perforin, and the membrane attack complex of the complement cascade to disrupt the membranes, but pathogenic microbes evolve complex cell walls and capsules to protect and then elaborate secretion systems to breach these barriers. These are but a few of the many mechanisms and counter-mechanisms that operate at the theater of war that is the plasma membrane. Similarly, genomic integrity is also an important battleground; therefore, the simplest of organisms have elegant DNA repair systems, high-fidelity replication enzymes, and restriction endonucleases. Yet viruses still integrate, and even counter by exploiting the rapid evolution afforded by replication errors. In turn, the adaptive immune system employs somatic hypermutation of antibody genes in a Darwinian germinal center reaction to hone high-affinity receptors against ever-shifting pathogen antigens.

Just as the plasma membrane and the genome are core components of life to be protected, exploited, and used in defense and offense, so is the pH of spaces large (stomach) and small (phagolysosome). It should come as no surprise that acids are used to great effect in signaling, killing, protecting, hiding, sampling, and evading by both host and microbe, and we should look to the trenches of protons and hydroxides for continued exploitation by both immune and pathogen systems.
